# Effects of Temperature and Blinking on Contact Lens Dehydration of Contemporary Soft Lens Materials Using an In Vitro Blink Model

**DOI:** 10.1167/tvst.10.8.11

**Published:** 2021-07-12

**Authors:** Vivian W. Y. Chan, Chau-Minh Phan, Hendrik Walther, William Ngo, Lyndon Jones

**Affiliations:** 1 Centre for Ocular Research & Education (CORE), School of Optometry and Vision Science, University of Waterloo, Waterloo, Ontario, Canada; 2 Centre for Eye and Vision Research (CEVR), Hong Kong

**Keywords:** contact lens, dehydration, in vitro, eye model, blinking

## Abstract

**Purpose:**

The purpose of this study was to evaluate the effects of temperature and blinking on contact lens (CL) dehydration using an in vitro blink model.

**Methods:**

Three silicone hydrogel (delefilcon A, senofilcon A, and comfilcon A) and two conventional hydrogel (etafilcon A and omafilcon A) CL materials were evaluated at 1 and 16 hours. The water content (WC) of the CLs was measured using a gravimetric method. Lenses were incubated on a blink model, internally heated to achieve a clinically relevant surface temperature of 35°C. An artificial tear solution (ATS) was delivered to the blink model at 4.5 µL/min with a blink rate of 6 blinks/min. A comparison set of lenses were incubated in a vial containing either 2 mL of ATS or phosphate-buffered saline (PBS) at 35°C.

**Results:**

Increasing temperature to 35°C resulted in a decrease in WC for all tested CLs over time (*P* ≤ 0.0052). For most CLs, there was no significant difference in WC over time between ATS or PBS in the vial (*P* > 0.05). With the vial system, WC decreased and plateaued over time. However, on the blink model, for most CLs, the WC significantly decreased after 1 hour but returned toward initial WC levels after 16 hours (*P* > 0.05).

**Conclusions:**

The reduction in WC of CLs on the eye is likely due to both an increase in temperature and dehydration from air exposure and blinking.

**Translational Relevance:**

This study showed that the novel, heated, in vitro blink model could be used to provide clinical insights into CL dehydration on the eye.

## Introduction

Contact lenses (CLs) have seen great adoption globally, with over 140 million wearers worldwide.^1^ Prior to commercialization, CLs must go through thorough testing of the device quality as well as ensuring the CLs are safe to use and will not change their parameters when worn. Some parameters that are tested include changes in lens dehydration,^2^^,^^3^ lens diameter,^4^ and lens thickness.^5^ These parameters can vary due to the nature of the CL material.^2^

Soft CLs are made of water-containing (hydrogel) materials and can be categorized as conventional hydrogels (CHs) or silicone hydrogels (SHs), depending on the composition of the material.^6^ Hydrogels are composed mainly of water held together by a crosslinking network of polymer side chains.^7^ The crosslinking network creates a scaffolding structure, which gives the material the ability to keep its shape but remain malleable to enable them to conform to the shape of the anterior ocular surface.^7^^,^^8^ The crosslinking structure allows for some permeation, such as oxygen transfer, making them an attractive material for manufacturing.^2^ As CLs are exposed to varying environmental conditions, the properties of the lens may undergo physical changes due to dehydration during in-eye wear.^2^^,^^7^

When CLs are worn, they are exposed to the environment and begin to dehydrate due to both evaporation^9^ and changes in temperature as they go from the blister pack or lens case (at room temperature [RT]) to ocular temperature (OT) once placed onto the eyes.^9^^,^^10^ As hydrogels dehydrate, the crosslinking structure begins to deform as empty spaces, previously occupied by water molecules, begin to form.^7^ Although lens dehydration may not have a direct effect on CL discomfort,^11^ the lens diameter,^4^ fit,^12^ and oxygen transmissibility^13^ may change due to dehydration, and as a result, lens comfort may decrease.^11^ As variability between patients and tear film composition differs greatly, CL dehydration may not have the same effect on all patients.^9^^,^^12^

Several studies have reported varying results on water content and dehydration under different conditions. Most studies agree that lens material composition impacts the dehydration of CLs, primarily due to their initial water content.^3^^,^^12^ However, the effects of environmental conditions, such as temperature and humidity, have varying results, with some studies reporting no significant difference^14^ and others reporting significant impacts on dehydration.^12^

Previous dehydration studies compared the effect of temperature on CL properties but submerged the lenses in saline, which does not explore the effects of tear film components and air exposure.^4^^,^^10^ Other studies directly compared in vitro and in vivo results, however, both studies had submerged their in vitro lenses in blister pack solutions at RT, whereas the in vivo lenses were exposed to tears, OT, and air from blinking.^2^^,^^15^ The current study used a blink model that incorporates an artificial tear solution and a blinking mechanism at OTs, in an attempt to provide more physiologically representative data.

The aim of this study was to evaluate the effects of temperature and blinking on the dehydration of CLs using an advanced in vitro blink model. This high throughput model allowed for results to be collected in a more controlled, physiologically relevant environment and showed potential to be used as a predictive tool for in vivo lens dehydration data, in addition to providing insights into CL dehydration on the eye.

## Methods

### Contact Lenses

Three commercially available SHs (delefilcon A, comfilcon A, and senofilcon A) and two CHs (etafilcon A and omafilcon A) lens materials were tested in this study (*n* = 5 for the vial system and *n* = 4 for the blink model). Of those, senofilcon A and etafilcon A were assessed in both daily disposable (DD) and reusable (RU) modalities. Thus, the total quantity of tested CLs equated to four SH and three CH materials. The properties of the CLs are listed in Table 1.

**Table 1.** Contact Lenses Materials Evaluated in the Study

USAN	Delefilcon A	Senofilcon A	Etafilcon A	Omafilcon A	Comfilcon A	Senofilcon A	Etafilcon A
**Commercial Name**	DAILIES TOTAL1	ACUVUE OASYS 1-DAY	1-DAY ACUVUE MOIST	Proclear 1 Day	Biofinity	ACUVUE OASYS	ACUVUE 2
**Manufacturer**	Alcon	Johnson & Johnson	Johnson & Johnson	Cooper Vision	Cooper Vision	Johnson & Johnson	Johnson & Johnson
**FDA classification**	V	V	IV	II	V	V	IV
**Water content (%)**	33	38	58	60	48	38	58
**Dk/t**	156.0	121.0	25.5	28.0	160.0	147.0	25.5
**Monomer composition**	Not disclosed	mPDMS, DMA, HEMA, siloxane macromere, PVP, TEGDMA	HEMA, MA	HEMA, PC	M3U, FMM, TAIC, IBM, HOB, NMNVA, NVP	mPDMS, DMA, HEMA, siloxane macromere, PVP, TEGDMA	HEMA, MA
**Wear modality**	DD	DD	DD	DD	RU	RU	RU
**Lens material**	SH	SH	CH	CH	SH	SH	CH

USAN, United States Adopted Name; mPDMS, monofunctional polydimethylsiloxane; DMA, *N*,*N*-dimethylacrylamide; HEMA, poly(2-hydroxyethyl methacrylate); PVP, polyvinyl pyrrolidone; TEGDMA, tetraethyleneglycol dimethacrylate; M3U, αω-bis(methacryloyloxyethyl iminocarboxy ethyloxypropyl)-poly(dimethylsiloxane)-poly(trifluoropropylmethylsiloxane)-poly(methoxy-poly(ethyleneglycol)propylmethylsiloxane; FMM, α-methacryloyloxyethyl iminocarboxyethyloxypropyl-poly(dimethylsiloxy)-butyldimethylsilane; TAIC, 1,3,5-triallyl-1,3,5-triazine-2,4,6(1H,3H,5H)-trione; IBM, isobornyl methacrylate; HOB, 2-hydroxybutyl methacrylate; NMNVA, N-methyl-N-vinyl acetamide; NVP, N-vinyl pyrrolidone; MA, methacrylic acid; PC, phosphorylcholine; DD, daily disposable; RU, reusable lenses; SH, silicone hydrogel; CH, conventional hydrogel.

The CLs selected for this experiment were chosen to represent different lens materials (CHs and SHs), varying modalities (DD and RU) and to encompass materials with varying water content values across the range typically used by clinicians. Etafilcon A and senofilcon A were chosen specifically to compare any differences within lens modality within the same lens material, as both are commercially available as DD and RU lenses. Delefilcon A was chosen due to its unique water gradient design, which has an SH-core and a CH-like surface.^16^

## Reagents

All materials were purchased from Sigma Aldrich (St. Louis, MO, USA) unless otherwise specified.

### Artificial Tear Solution

The artificial tear solution (ATS) used in this study was previously described by our group.^17^^–^^19^ The ATS contains various salts, proteins, and lipids (see Table 2).

**Table 2.** Artificial Tear Solution Components

Salts (mg/mL)	Lipids (mg/mL)	Proteins (mg/mL)
NaCl (5.26)	Oleic acid (0.0018)	Mucin (0.15)
KCl (1.19)	Oleic acid methyl ester (0.012)	Albumin (0.20)
Na_3_C_6_H_5_O_7_ (0.44)	Triolein (0.016)	Lysozyme (1.90)
Glucose (0.036)	Cholesterol (0.0018)	Lactoferrin (1.80)
Urea (0.072)	Cholesteryl oleate (0.024)	
CaCl_2_ (0.07)	Phosphatidylcholine (0.0005)	
Na_2_CO_3_ (1.27)		
KHCO_3_ (0.30)		
Na_2_HPO_4_ (3.41)		
HCl (0.94)		
ProClin 300 (200 µL/L of solution)		

### Experimental Design for Vial Incubation

Lenses were incubated in vials with 2 mL of incubation solution, either phosphate buffered saline (PBS) or ATS. At RT (22°C ± 2°C), lenses were placed on an orbital shaker at 60 rotations per minute for 1 or 16 hours. To mimic OT,^20^^–^^22^ lenses were placed in a shaking incubator (New Brunswick Innova, Marshall Scientific, Hampton, NH, USA) at 35°C for 1 or 16 hours. After the incubation period, lenses were removed from the vials and water content was measured.

### Synthesis of Eyeball and Eyelid for Blink Model

The procedures for the synthesis of the eyelids of the blink model were adopted from methods described by Hyon et al.^23^ In brief, polyvinyl alcohol (PVA) was added to a mixture of dimethyl sulfoxide and Milli-Q water (8:2) to achieve a concentration of 15% weight/volume (w/v). The mixture was then stirred and heated at 120°C for 1 hour before being poured into a mold. The molds were then stored at -20°C for 24 hours for gelation. After the eyelids gelled, they were removed from the molds and placed in 500 mL of Milli-Q water, renewed daily for 3 days to remove any residual dimethyl sulfoxide.

The eyeball structure was designed using computer-aided design (CAD) software and 3D-printed (Photon S, Anycubic, Shenzhen, China) with SLA resin as a four-piece hollow structure. This design allowed heated water to be pumped through the model to achieve and simulate an ocular surface temperature of 35°C ± 0.8°C. The surface of the eyeball was coated with silicone material to limit the absorption of tear film components on the eye model, as shown in Figures 1A and 1B. Furthermore, the silicone polymer does not absorb water from the contact lenses, which could potentially exacerbate any measured dehydration.

**Figure 1A.**
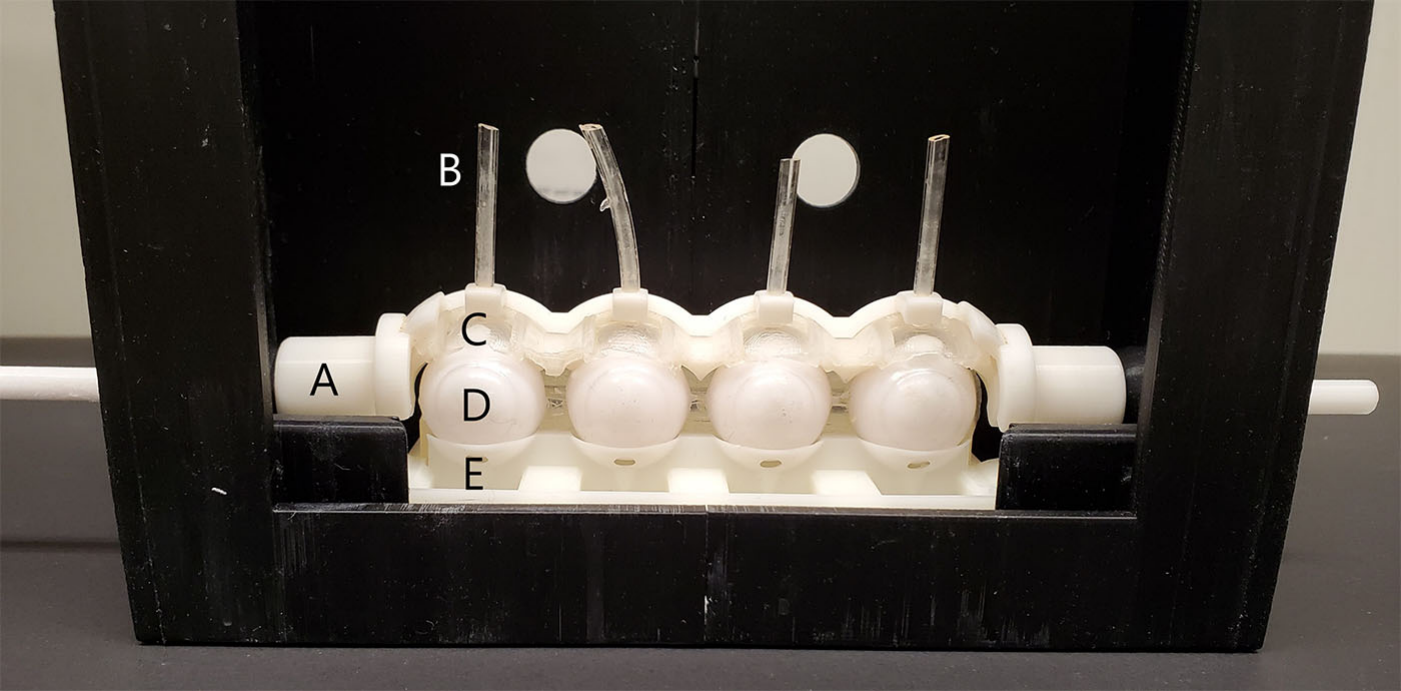
In vitro eye blink model used in this study. (**A**) Connector from eyelid to blink motor. (**B**) Tubing for artificial tear fluid. (**C**) Eyelid. (**D**) Silicone eyeball. (**E**) Lower eyelid with trough to hold excess tear fluid.

**Figure 1B.**
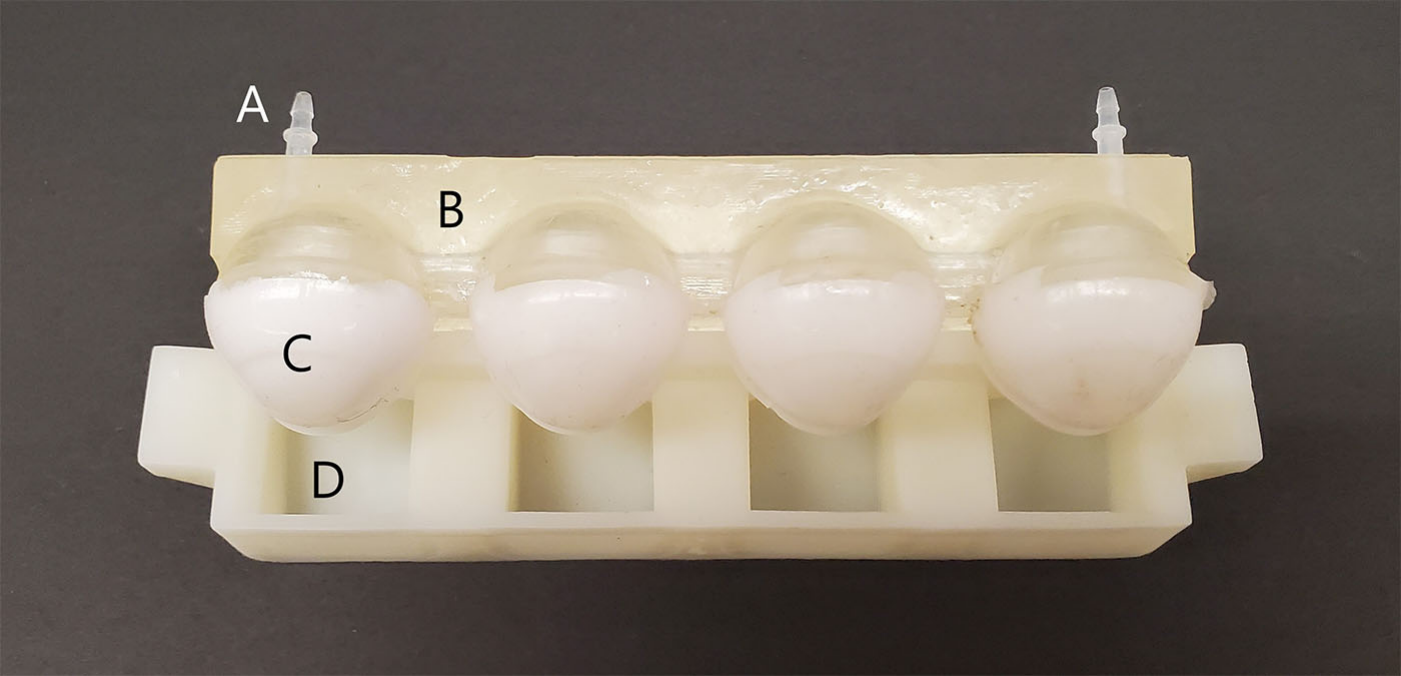
Top view of eyeball structure. (**A**) Inlet and outlet for heated water. (**B**) Hollow eyeball structure to allow heated water through. (**C**) Silicone eyeball. (**D**) Lower eyelid with trough to hold excess tear fluid.

### Set-Up of Blink Model

The set-up for the blink model is shown in Figure 1A and Figure 1B. The blink motion and blink rate were controlled by an Arduino board (WEMOS D1 R2 Wifi ESP8266) and attached motor (Adafruit Industries, New York, NY, USA). The blink rate was set to six blinks/min. Tubing was attached to the top of an eyelid support structure, which was then connected to a commercial microfluidic pump (PHD Ultra, Harvard Apparatus, Holliston, MA, USA) to deliver ATS to the system. In preliminary trials, flow rates close to reported tear flow values of 1 µL/min^24^ were insufficient to maintain a reliable flow rate of fluid on the eyes. We hypothesize that the increase in temperature caused an increase in evaporation rates, which caused the CLs to shrink on the blink model. Through trial and error, an appropriate tear flow rate of 4.5 µL/min was chosen for this experiment. A secondary pump (Auto Dosing pump, Jebao, Guangdong, China) administered 40°C water from a water bath (Aquasonic Model 50D; VWR International, Radnor, PA, USA) through the back of the eyeballs to achieve a surface temperature of 35°C. A video demonstrating surface heating of the eyeball is provided as Supplementary Video S1. The blink model was placed in a humidity chamber to maintain an average temperature and humidity of 23.6°C ± 1.6°C and 91.8% ± 3.2%, respectively.

### Experimental Design for Blink Model

The blink model was equilibrated for 30 minutes prior to the addition of CLs to ensure adequate tear flow on the eyelid and eyeball. The lenses were removed from the blister pack and placed directly on the blink model. After 1 or 16 hours, the lenses were removed, and water content measured. Time points of 1 and 16 hours of lens incubation were chosen to explore the immediate and extended effect of lens dehydration for a daily disposable CL worn across a typical day.

### Water Content Determination

The procedure to measure content from the CLs was adopted from Jones et al.^12^ In brief, lenses were removed from their blister pack or vial incubation and gently blotted on lens paper to remove any excess solution on the lens surface. Once blotted, the lens was placed on a digital balance (Sartorius M100, Göttingen, Germany), and the wet weight recorded. Lenses were then heated at 105°C for 1 hour and then cooled in a desiccator for 30 minutes before weighed again to determine the dry weight. The two measurements were then used to determine the water content of the lens from the blister pack and from each individual time point using Equation 1.

(1) $$\;\;\;\begin{array}{c} \%\;Water\;content=\left(\frac{wet\;weight-dry\;weight}{wet\;weight}\right)\times 100 \end{array}$$

### Relative Dehydration

Determination of relative dehydration was adopted from Jones et al.^12^ In brief, using the water content values calculated from Equation 1, the relative dehydration was determined using Equation 2. Initial equilibrium water content (EWC) was determined from the CL taken out of the blister pack, and the final EWC was determined after incubation on the blink model or in the vial.

(2) $$\;\;\;\begin{array}{c} Relative\;\%\;dehydration=\left(\frac{initial\;EWC\;-final\;EWC}{initial\;EWC}\right)\times 100 \end{array}$$

### Statistical Analysis

Statistical analysis and graphs were plotted using GraphPad Prism version 8 software (GraphPad, La Jolla, CA, USA). All data are expressed as a percentage in mean ± SD. With the vial system, a 2-way ANOVA with a post hoc Sidak multiple comparison test was used to test the differences in water content between time and incubation temperature for both PBS and ATS conditions. A second 2-way ANOVA with a post hoc Sidak multiple comparison test was used to test the differences in water content between time and incubation solution for both RT and OT conditions.

Unpaired *t*-tests between lens materials were used to test the difference in water content for both the vial and blink model systems. A 2-way ANOVA with a post hoc Sidak multiple comparisons test was used to test the differences in water content between lens material and time for both the vial and blink model. A 2-way ANOVA with a post hoc Sidak multiple comparisons test was used to test the differences in water content between lens material and model systems, for both 1 and 16 hours. Statistical significance was achieved at the level of *P* < 0.05.

## Results

### Water Content From Vial Incubation

The water content of all tested CLs in PBS and ATS over time at both RT (22°C) and OT (35°C) in the vial system is summarized in Table 3.

**Table 3.** Equilibrium Water Content of Various Lens Materials Measured After Vial Incubation in Two Test Solutions and at Two Temperatures

	PBS				ATS			
	EWC (%) at RT (Mean ± SD)		EWC (%) at OT (Mean ± SD)		EWC (%) at RT (Mean ± SD)		EWC (%) at OT (Mean ± SD)	
Contact Lens Material NWC (*n* = 5 Each)	1 hr	16 hr	1 hr	16 hr	1 hr	16 hr	1 hr	16 hr
**Delefilcon A** 33% SH DD	34.48 ± 0.00	34.48 ± 0.00	29.09 ± 1.21	29.09 ± 1.21	29.55 ± 2.61	34.01 ± 1.05	28.55 ± 1.48	29.05 ± 2.20
**Senofilcon A** 38% SH DD	36.32 ± 1.93	35.17 ± 1.09	32.63 ± 2.31	32.26 ± 0.00	35.94 ± 1.62	34.38 ± 0.00	32.23 ± 1.55	30.84 ± 2.57
**Etafilcon A** 58% CH DD	53.01 ± 0.72	52.69 ± 0.88	50.00 ± 0.00	46.95 ± 1.09	54.54 ± 0.67	55.12 ± 0.63	53.33 ± 0.00	50.69 ± 0.94
**Omafilcon A** 60% CH DD	59.62 ± 0.55	59.38 ± 0.00	58.33 ± 0.59	58.06 ± 0.00	58.06 ± 0.00	58.59 ± 0.72	57.78 ± 0.63	57.23 ± 0.77
**Comfilcon A** 48% SH RU	48.06 ± 2.45	48.89 ± 1.01	47.32 ± 1.86	46.52 ± 1.73	49.23 ± 1.72	49.63 ± 0.83	46.12 ± 1.47	47.69 ± 2.11
**Senofilcon A** 38% SH RU	35.71 ± 0.00	35.71 ± 0.00	30.77 ± 0.00	30.77 ± 0.00	35.71 ± 0.00	34.76 ± 1.30	30.77 ± 0.00	30.77 ± 0.00
**Etafilcon A** 58% CH RU	55.61 ± 0.60	55.06 ± 1.16	52.52 ± 0.83	52.82 ± 0.68	53.69 ± 0.78	54.81 ± 0.60	51.61 ± 0.00	47.54 ± 1.01

NWC, nominal water content; PBS, phosphate buffered saline; ATS, artificial tear solution; EWC, equilibrium water content; SH, silicone hydrogel; CH, conventional hydrogel; DD, daily disposables; RU, reusable. RT, room temperature, 22°C ± 2°C; OT, ocular temperature 35°C ± 0.8°C.

### Lens Water Content as a Function of Temperature (Vial) and Time

Overall, lower water content was observed for OT compared to RT. For all lenses, this difference in water content was statistically significant (*P* ≤ 0.0052), except omafilcon A and delefilcon A at 1 hour in ATS (*P* > 0.05). Water content for comfilcon A was only significantly lower after 1 hour in ATS (*P* = 0.0029).

### Lens Water Content as a Function of Incubation Solution (Vial) and Time

For most lenses tested, there were no significant differences in water content due to incubation solution. There was no significant difference for senofilcon A (DD) or comfilcon A for either incubation solution or time (*P* > 0.05). For delefilcon A, there was no significant difference except for 1 hour incubation at RT (*P* < 0.0001), where PBS incubation had a higher water content than ATS incubation. For senofilcon A (RU), there was no significant difference except for 16 hours of incubation at RT (*P* = 0.0277), where PBS incubation had a higher water content than ATS incubation. Etafilcon A (RU) showed significant differences RT incubation at 1 hour (*P* = 0.0004) and OT incubation for 16 hours (*P* < 0.0001), both with a higher water content value in PBS over ATS incubation. Omafilcon A showed significant differences for all conditions except 1 hour incubation at OT (*P* ≤ 0.0277), all with a higher water content value in PBS over ATS incubation. Etafilcon A (DD) showed a significant difference for all conditions (*P* ≤ 0.0014), with a higher water content value for ATS over PBS incubation.

### Lens Water Content as a Function of Lens Material

An unpaired *t*-test between SH and CH lens materials showed a significant difference in water content for both vial (*P* = 0.0296) and blink model (*P* = 0.0135) incubation systems. Both systems showed a higher mean water content value for CH lens materials (51.82% vial and 51.09% blink system) compared to SH lens materials (34.59% vial and 34.89% blink system) when incubated in ATS over 16 hours at OT.

### Lens Water Content on Blink Model

**Figure 2.**
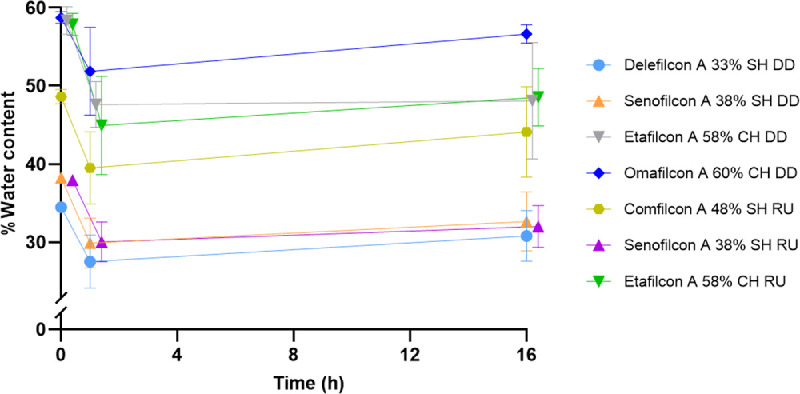
Percent water content of contact lenses over time on blink model system with artificial tear solution at ocular temperature (35°C). SH, silicone hydrogel; CH, conventional hydrogel; DD, daily disposables; RU, reusable; error bars represent standard deviation.

The water content of all tested CLs on the blink model over time at ocular temperature (35°C) is shown in Figure 2 and summarized in Table 4.

**Table 4.** Equilibrium Water Content of Various Lens Materials Measured After Blink Model Incubation at OT With ATS Over Time

Blink Model (n = 4)	Delefilcon A (DD)	Senofilcon A (DD)	Etafilcon A (DD)	Omafilcon A (DD)	Comfilcon A (RU)	Senofilcon A (RU)	Etafilcon A (RU)
**EWC (%) after 1 hour (Mean ± SD)**	27.50 ± 3.39	29.89 ± 3.20	47.61 ± 2.94	51.85 ± 5.63	39.50 ± 4.62	30.03 ± 2.56	44.95 ± 6.31
**EWC (%) after 16 hours (Mean ± SD)**	30.79 ± 3.25	32.65 ± 3.77	48.08 ± 7.46	56.64 ± 1.18	44.10 ± 5.79	32.00 ± 2.67	48.55 ± 3.69

OT, ocular temperature 35°C ± 0.8°C; ATS, artificial tear solution; EWC, equilibrium water content; DD, daily disposables; RU, reusable.

### Lens Water Content as a Function of Lens Material and Time

All lenses showed a significant decrease in water content after 1 hour of incubation in the vial system compared to the blister pack (*P* ≤ 0.0254). After 16 hours of incubation in the vial system, only comfilcon A did not show a significant decrease in water content (*P* > 0.05).

All lenses showed a significant decrease in water content after 1 hour of incubation on the blink model compared to the blister pack (*P* ≤ 0.0266). Both etafilcon A lens modalities showed an additional significant decrease in water content after 16 hours of incubation on the blink model (*P* = 0.0004 for DD and *P* = 0.0016 for RU). After 16 hours of incubation with ATS on the blink model, etafilcon A had the greatest decrease in water content (10.30% ± 7.46), and omafilcon A had the least decrease of water content (2.08% ± 1.18).

### Change in Water Content Between Vial Incubation and Blink Model

After 1 hour of incubation at OT in ATS, there was a significant difference in water content between the two incubation systems for all lenses (*P* ≤ 0.0364) except delefilcon A, and senofilcon A (DD and RU). All lenses had a higher water content in the vial system compared to the blink model. After 16 hours of incubation at OT in ATS, there were no significant differences in water content between the two incubation systems for any of the CLs tested.

A comparison of water content between the two incubation systems allows for the determination of how much change in water content is due to certain factors. The difference in water content between RT and OT incubation in ATS in the vial system determines the change in water content due to heating alone. The difference in water content between the blink model and vial system at OT determines the change in water content due to other factors that are not due to heating. The results are summarized in Table 5.

**Table 5.** Water Content After 16 Hours of Incubation in ATS

**Vial Model (*n* = 5)**	**Delefilcon A (DD)**	**Senofilcon A (DD)**	**Etafilcon A (DD)**	**Omafilcon A (DD)**	**Comfilcon A (RU)**	**Senofilcon A (RU)**	**Etafilcon A (RU)**
EWC (%) at RT(Mean ± SD)	34.01 ± 1.05	34.38 ± 0.00	55.12 ± 0.63	58.59 ± 0.72	49.63 ± 0.83	34.76 ± 1.30	54.81 ± 0.60
EWC (%) at OT(Mean ± SD)	29.05 ± 2.20	30.84 ± 2.57	50.69 ± 0.94	57.23 ± 0.77	47.69 ± 2.11	30.77 ± 0.00	47.54 ± 1.01
**Blink model (*n* = 4)**	**Delefilcon A (DD)**	**Senofilcon A (DD)**	**Etafilcon A (DD)**	**Omafilcon A (DD)**	**Comfilcon A (RU)**	**Senofilcon A (RU)**	**Etafilcon A (RU)**

EWC (%) at OT (Mean ± SD)	30.79 ± 3.25	32.65 ± 3.77	48.08 ± 7.46	56.64 ± 1.18	44.10 ± 5.79	32.00 ± 2.67	48.55 ± 3.69
**Change in water content**							

Due to heating in a vial(∆EWC between RT and OT)	−4.97 ± 2.20	−3.53 ± 2.57	−4.43 ± 0.94	−1.36 ± 0.77	−1.94 ± 2.11	−3.99 ± 0.00	−7.28 ± 1.01
Due to heating and air exposure on blink model(∆EWC between blink model at OT and vial at RT)	−3.22 ± 3.25	−1.73 ± 3.77	−7.04 ± 7.46	−1.95 ± 1.18	−5.53 ± 5.79	−2.76 ± 2.67	−6.26 ± 3.69

ATS, artificial tear solution; EWC, equilibrium water content; RT, room temperature; OT, ocular temperature; DD, daily disposables; RU, reusable.

### Relative Percent Dehydration

**Figure 3.**
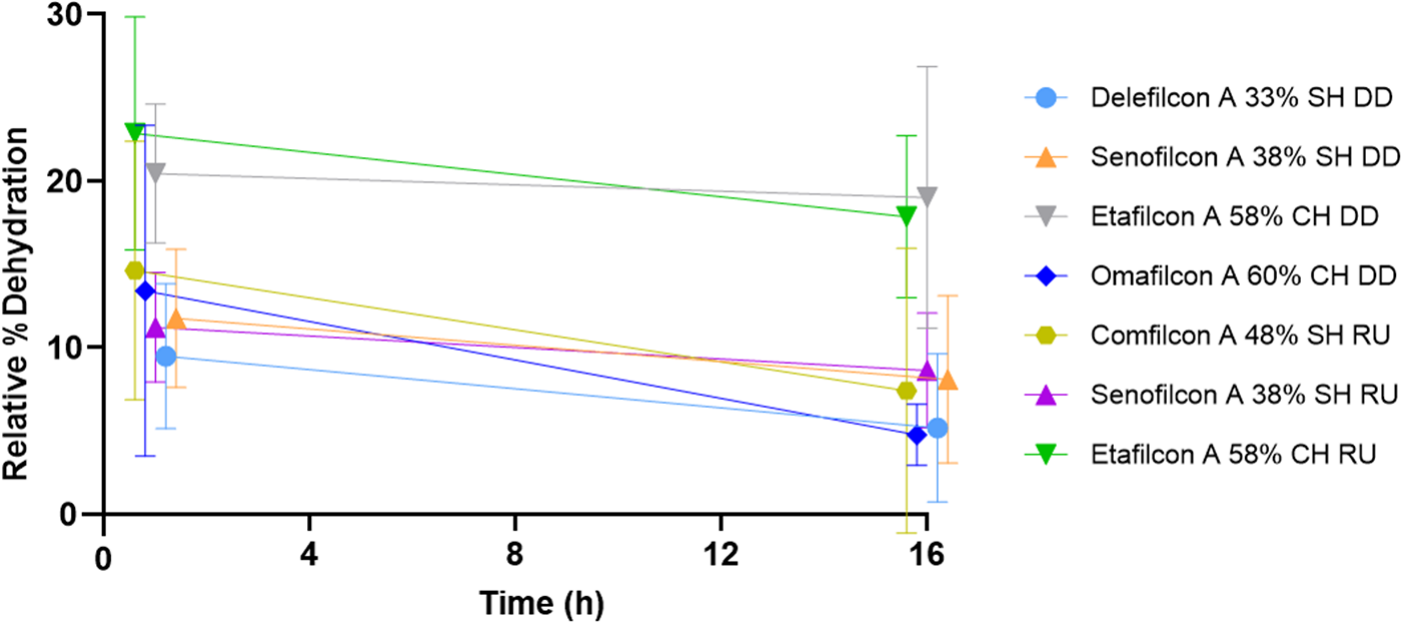
Relative percent dehydration of contact lenses over time on blink model with artificial tear solution at ocular temperature (35°C). SH, silicone hydrogel; CH, conventional hydrogel; DD, daily disposables; RU, reusable; error bars represent standard deviation.

Figure 3 shows the relative percent dehydration of the tested CLs on the blink model at OT. At 1 hour, delefilcon A had the lowest amount of relative dehydration and etafilcon A (RU) had the highest amount of relative dehydration. At 16 hours, omafilcon A showed the lowest amount of relative dehydration, and etafilcon A (DD) showed the highest amount of relative dehydration. However, no statistical significance was determined.

## Discussion

This study examined the effects of incubation solution and temperature on the water content of several contemporary CL materials. In addition, the study also examined the dehydration of CLs using an advanced in vitro blink model. To summarize, an increase in temperature was accompanied by a decrease in water content of all tested CLs. For most CLs, vial incubation solution did not have an effect on water content. Over time, the water content of CLs incubated in the vial model plateaued, whereas with the blink model, there was recovery in water content after 16 hours of incubation.

### Water Content From Vial Incubation

The results from this study support previously published studies^2^^,^^12^^,^^14^ on the lens material being a significant contributing factor to differences in CL water content. Initial water content plays an important role in the loss of water in CL materials.^2^ The water in polymer material can be categorized as free water, loosely bound water, or tightly bound water.^2^^,^^25^ Free water in the polymer material does not interact with the polymer, and dehydration of free water occurs quickly.^2^ Tightly bound water directly interacts with the polar side chains of the polymer material through hydrogen bonding, in which dehydration of tightly bound water is unlikely to occur at room temperature.^2^ Loosely bound water in the polymer material is in a state between the free and tightly bound water forms, where they interact with the polar side chains of the polymer material but are more strongly associated with water molecules via hydrogen bonding.^25^ CLs with higher nominal water content have a greater amount of loosely bound water.^12^^,^^25^ As a result, the rate of dehydration for high water content CLs is often greater and occurs more rapidly.^3^^,^^12^^,^^13^^,^^26^^,^^27^ This was demonstrated in this study, with both etafilcon A lens modalities with the second highest nominal water content, 58%, having the highest relative percent dehydration (see Figure 3). However, omafilcon A, the lens material with the highest nominal water content in this study at 60%, did not show a high dehydration rate due to the presence of phosphorylcholine, demonstrating that the lens material does play a role in dehydration of CLs, as reported in literature.^2^^,^^27^^,^^28^ In addition, the hydrophobicity of different lens materials plays a role in the strength of hydrogen bonding to water molecules in the hydrogel.^25^ As a result, each lens material will have different water-binding capabilities and water content, and consequently will have different rates of dehydration.

However, the hydrophobicity of the lens material is not the only factor affecting lens water content and dehydration rates. The solution in which the lens resides, temperature, and air exposure may also play a role in the dehydration rates of CL materials.^25^

### Change in Water Content Due to Temperature

Based on the results of the vial study, a change in temperature from RT (22°C) to OT (35°C), causes a reduction in water content for all the CLs tested in this study. This phenomenon has been previously reported^9^^,^^10^ and may be due to different lens polymer configurations, which may cause water to be expelled at different rates.^2^ The change in temperature can cause the hydrogel matrix to contract and shrink, causing the expulsion of water molecules within the hydrogel matrix.^7^ This study demonstrated that the components of the CL materials likely have an effect on the rate of dehydration. For example, omafilcon A had the highest water content, but did not show a rapid dehydration rate in comparison to other high water content materials. Omafilcon A contains phosphorylcholine, which has a high affinity for water, as reported in literature,^2^^,^^27^^,^^28^ which consequently leads to a slow dehydration profile.

### Change in Water Content Due to Incubation Solution and Lens Material

The lens materials may behave differently when incubated in distilled water, PBS,^25^ or ATS.^29^ Salts and proteins in the surrounding solution can interact with the polymer side chains^29^ as well as cause the lens material to swell.^2^ Comparing the lens materials after the 16 hours of incubation, in both solutions, showed no statistically significant difference between the incubation solutions (*P* > 0.05) for most lenses. However, the CH lenses in this study, etafilcon A (DD) and omafilcon A, showed a significant difference between both incubation solutions when incubated at OT for 16 hours (*P* < 0.0001 and *P* = 0.0277, respectively), whereas etafilcon A (RU) showed a significant difference between incubation solutions when incubated for 16 hours at OT only (*P* < 0.0001). As the amount of bound water is expected to be similar in both high and low water content lenses, the amount of free water is greater for high water content CLs.^25^ Etafilcon A is a lens material that has been previously shown to have high dehydration rates driven by osmolarity due to its ionic lens material and high water content.^3^^,^^12^^,^^26^^–^^28^ In this study, etafilcon A (DD) has lower water content in PBS whereas etafilcon A (RU) has lower water content in ATS after 16 hours of incubation at OT. It is interesting, but unclear, why the two different modalities of the same lens material had different dehydration patterns when incubated in the two solutions.

Omafilcon A has previously been shown to have a lower dehydration rate relative to other high water content CLs.^14^^,^^27^^,^^28^ It has been proposed that this is due to the presence of phosphorylcholine, which has a high affinity to water.^2^^,^^27^^,^^28^ As the ATS is a more complex solution with several components, it is not surprising to see omafilcon A with higher water content in PBS compared to ATS after 16 hours of incubation at OT.

### Water Content Determination Using the Blink Model

The blink model was used to evaluate the change in water content of CLs as a function of both time and lens material. The blink model incorporates the use of ATS, OT, and a blink mechanism to best mimic physiological conditions. After 1 hour of incubation on the blink model, all lenses showed a statistically significant decrease in water content (*P* ≤ 0.0266). However, after 16 hours of incubation, most CLs recovered their water content to blister pack values, with no statistically significant difference (*P* > 0.05). It is hypothesized that the combination of high humidity levels (>90%) and the blink mechanism causes additional movement of the polymers in the CL material when compared to the vial system.^2^ This may allow for greater water binding over time. The only CLs that continued to show a significant decrease in water content after 16 hours, were both etafilcon A lens materials (DD: *P* = 0.0004; and RU: *P* = 0.0016).

Additionally, after 16 hours, etafilcon A showed the largest overall decrease in water content with the blink model (10.30% ± 7.46%). These results are in agreement with previously published studies on ionic, high water content CLs having a greater and faster dehydration rate compared to low water content CLs.^3^^,^^12^^,^^26^^–^^28^ In contrast, omafilcon A had the lowest overall decrease in water content on the blink model (2.08% ± 1.18%). This agrees with published studies based on the presence of phosphorylcholine of omafilcon A. The high affinity for water of this lens material shows a different dehydration pattern to other high water content CLs.^2^^,^^27^^,^^28^ Both in vitro^2^^,^^12^ and in vivo^2^^,^^27^^,^^28^ studies have previously shown these results and demonstrate that this in vitro blink model is an excellent model to use when predicting dehydration patterns.

### Change in Water Content Over Time Between Vial Incubation and Blink Model

**Figure 4.**
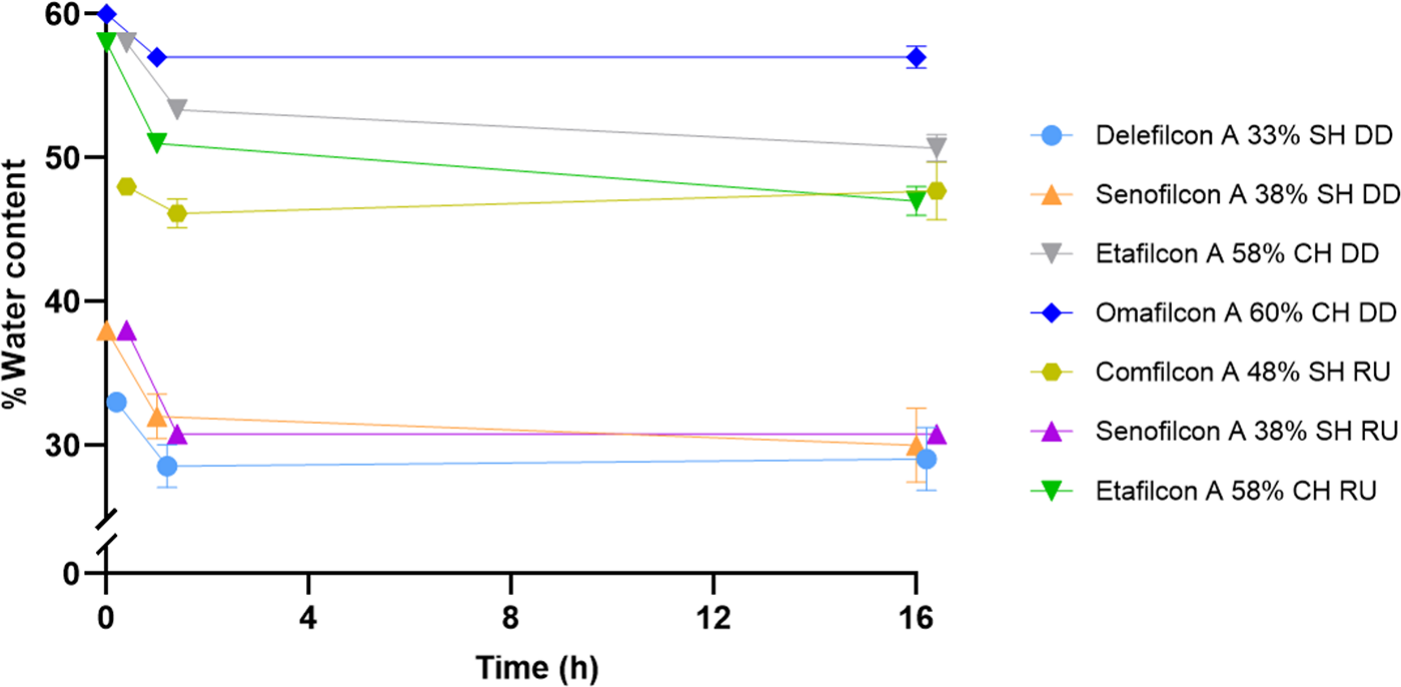
Percent water content of contact lenses over time in vial system with artificial tear solution at ocular temperature (35°C). SH, silicone hydrogel; CH, conventional hydrogel; DD, daily disposables; RU, reusable; error bars represent standard deviation.

On both incubation systems, the CLs showed a statistically significant decrease in water content from the blister pack after 1 hour of incubation in ATS at OT (*P* ≤ 0.0266). With vial incubation, all the CLs, except comfilcon A (*P* > 0.05), showed a statistically significant decrease in water content after 16 hours of incubation compared to the blister pack (*P* ≤ 0.0004). However, with the blink model system, only the etafilcon A lens modalities (DD: *P* = 0.0004; and RU: *P* = 0.0016) showed a significant decrease in water content at 16 hours of incubation, whereas all other changes in water content of the other CL materials were not statistically different (*P* > 0.05). When the lens materials were incubated in the vial system, CLs demonstrate a plateau of dehydration (Figure 4), whereas with the blink model, CLs show signs of recovering water content after 16 hours (see Figure 2). We suspect that the blinking mechanism allows replenishment of the tear film, similar to the physical reflex of increased blinking during dry eye symptoms, which prolongs the time to CL dehydration, allowing a slight recovery for the CLs on the blink model. It is also hypothesized that the increased humidity levels working together with the blinking mechanism may increase the movement of the polymers of the CL materials, allowing for greater water binding over time. The vial CLs may have reached a saturation point as the lenses are fully immersed in solution, as shown in previously published studies.^19^ Due to the high humidity (>90%) on the blink model, we suspect that the blink model lenses also approach their saturation level, but delayed due to the increased humidity. A high humidity value was chosen for this study to test the effects of other variables without the addition of low humidity, which has previously been shown to change water content values.^9^^,^^12^ Compared to in vivo studies, we would expect much lower water content values due to lower humidity levels. Further investigation with varying humidity is required to explore these results.

Between incubation systems, at 1 hour, there were only significant differences between the vial system and blink model for both etafilcon A lens modalities, omafilcon A, and comfilcon A. After 16 hours of incubation, no significant differences were seen between the two incubation systems.

By comparing the change in water content between the two incubation systems, the percent change due to heating was determined and summarized in Table 5. The change in water content from the vial incubation was only due to the heating of the incubation solution. The differences in water content between the vial incubation and the blink model may be due to air exposure from the blink mechanism of the blink model that could further cause varying degrees of deposition, shear stress, and other additional interactions that have not been investigated in this study. For the majority of the tested CLs, the results of this study demonstrate that the amount of water loss can largely be attributed to the increase in temperature of the incubation solutions alone. For etafilcon A (DD) and comfilcon A, the amount of water loss is mainly due to a combination of heating, dehydration, and air exposure. As from previously published studies,^3^^,^^12^^,^^13^ high water content lens materials tend to have higher rates of dehydration compared to CLs with low water content. Further work on the blink model will be required to determine which additional factors cause a change in water content of lens materials.

### Clinical Significance

Water content and dehydration of CLs have varying results regarding its correlation to comfort. Some studies report that CL dehydration plays a major role in the production of reduced comfort,^12^ whereas others report no correlation.^11^^,^^14^ However, several studies agree that further testing is required to better understand the effect of CL dehydration on lens comfort.^2^^,^^12^^,^^14^ This study attempted to determine the loss in water content due to temperature as well as dehydration over time. For some of the CLs tested in this study, half or more of the CL dehydration was due to heating the material to ocular temperature. For other CLs, temperature had a lower effect on dehydration. The factors that would have caused lens dehydration besides heating could be lens polymer composition, air exposure, shear stress from the blinking mechanism, or other factors that remain to be determined. Dehydration observed in the first hour of incubation in both in vitro models do not reflect the discomfort scores in in vivo studies,^30^^,^^31^ suggesting that dehydration may not have a direct association with end of day discomfort. This is further supported as the water content after 16 hours of incubation on the blink model recovers to initial blister pack water content values.

A better understanding of how lens materials react due to changes in temperature can help clinicians determine the lens materials which are best suited for certain consumers. This study shows that higher temperatures will lead to a decrease in water content in all lens materials. Certain CL materials that have better retention of their water content, therefore, may be better suited for use in hotter climates. It has been previously published that the dehydration of some lens materials can affect lens fit^12^ and oxygen transmissibility,^13^ resulting in discomfort and potentially other clinical problems. As a result, having a better understanding of CL dehydration allows for more informed choices in lens materials and subsequently, lens comfort.

Overall, the average dehydration of all test lenses combined was 5.91% ± 4.79% after 16 hours of incubation when using the blink model. Comparatively, two clinical studies^32^^,^^33^ showed that the decrease in water content of DD CLs was less than 5% after 12 hours of wear. We suspect that our results should differ from in vivo data as this study was conducted at a humidity level of >90%, and varying humidity levels have previously been shown to affect dehydration rates.^9^^,^^12^ At lower humidity levels, a higher rate of dehydration is expected. However, it is unclear whether or not a 5% change in water content is enough to cause dryness or discomfort for CL wearers, therefore, further testing is required. Based on the results from this study, there is variability seen between all the CLs tested, warranting further testing in addition to comparison to in vivo data. By using more advanced in vitro models and changing the parameters, more information on CL dehydration can be explored.

## Conclusion

In conclusion, this study compared the effects of incubation temperature, incubation time, and incubation solution on CL dehydration of various lens materials in both a vial system and an advanced in vitro blink model. Increased temperature resulted in a decrease in water content for all CLs. Furthermore, with the novel features of the in vitro blink model, when lenses were exposed to air exposure during blinking, CL dehydration was observed. However, the majority of the reduction in water content occurred within the first hour and did not reduce further over time. For most tested lenses, the type of incubation solution did not have an effect on water content. CL materials, however, played a major role in differences in water content. A novel finding with the blink model was a recovery in water content demonstrated after 16 hours of incubation. Further work is required to investigate this phenomenon. Overall, by examining the water content values, the amount of dehydration due to change in temperature could be determined with the two incubation systems. Generally, using more advanced in vitro models that better mimic the ocular environment in vitro will provide more representative data to translate laboratory results to real world CL wear scenarios.

## Supplementary Material

Supplement 1

## Supplementary Material

**Supplementary Video S1**. Thermograph time lapse of front surface of an eyeball heated to ocular temperature (35°C, right) compared to control eyeball without heating (19°C, left).
